# BCL9L expression in pancreatic neoplasia with a focus on SPN: a possible explanation for the enigma of the benign neoplasia

**DOI:** 10.1186/s12885-016-2707-1

**Published:** 2016-08-18

**Authors:** Cora Hallas, Julia Phillipp, Lukas Domanowsky, Bettina Kah, Katharina Tiemann

**Affiliations:** Institut für Hämatopathologie, Fangdieckstr. 75, Hamburg, 22547 Germany

**Keywords:** Solid pseudopapillary neoplasms of the pancreas, Intraductal papillary mucinous neoplasm, Pancreatic adenocarcinoma, FLI1, BCL9L, INPP5D, IGFBP3

## Abstract

**Background:**

Solid pseudopapillary neoplasms of the pancreas (SPN) are rare tumors affecting mainly women. They show an activating mutation in CTNNB1, the gene for β-catenin, and consequently an overactivation of the Wnt/β-catenin pathway. This signaling pathway is implied in the pathogenesis of various aggressive tumors, including pancreatic adenocarcinomas (PDAC). Despite this, SPN are characterized by an unusually benign clinical course. Attempts to explain this lack of malignancy have led to the discovery of an aberrant expression of the transcription factor FLI1 in SPN.

**Methods:**

In 42 primary pancreatic tumors the RNA-expression of the FLI1 targets DKK1, INPP5D, IGFBP3 and additionally two members of the Wnt/β-catenin pathway, namely BCL9 and BCL9L, was investigated using quantitative real time PCR. Expression of these genes was evaluated in SPN (*n* = 18), PDAC (*n* = 12) and the less aggressive intraductal papillary mucinous neoplasm IPMN (*n* = 12) and compared to normal pancreatic tissue. Potential differences between the tumor entities were evaluated using students *t*-test.

**Results:**

The results demonstrated a differential RNA-expression of BCL9L with a lack of expression in SPN (*p* < 0.001), RNA levels similar to normal tissue in IPMN and increased expression in PDAC (*p* < 0.04). Further, overexpression of the cyclin D1 inhibitor INPP5D in IPMN (*p* < 0.0001) was found. PDAC, on the other hand, showed the highest expression of IGFBP3 (*p* < 0.00001) with the gene still being significantly overexpressed in IPMN (*p* < 0.001). Nevertheless the difference in expression was significant between PDAC and IPMN (*p* < 0.05) and IGFBP3 RNA levels were significantly higher in PDAC and IPMN than in SPN (*p* < 0.0001 and *p* < 0.02, resp.).

**Conclusions:**

This study demonstrates a significantly decreased expression of the β-catenin stabilizing gene BCL9L in SPN as a first clue to the possible reasons for the astonishingly benign behavior of this entity. In contrast, high expression of the gene was detected in PDAC supporting the connection between BCL9L expression and tumor malignancy in pancreas neoplasias. IPMN, accordingly, showed intermediate expression of BCL9L, but instead demonstrated a high expression of the cyclin D1 inhibitor INPP5D, possibly contributing to the better prognosis of this neoplasia compared to PDAC.

**Electronic supplementary material:**

The online version of this article (doi:10.1186/s12885-016-2707-1) contains supplementary material, which is available to authorized users.

## Background

Solid pseudopapillary neoplasms of the pancreas (SPN) are rare tumors affecting women in the overwhelming majority of cases. 90–95 % of these neoplasms are clinically benign and only few cases show malignant growth with metastases in liver and mesenterium. At the molecular level SPN are defined by a mutation in exon 3 of CTNNB1, the gene for β-catenin, found in about 90 % of cases [1]. β-catenin is part of the Wnt signaling pathway and plays a crucial role in embryonal development, but its deregulated activity has been implicated in the pathogenesis of a variety of cancers [2] and specifically in pancreatic cancer [3]. The activating mutation of CTNNB1 in SPN is often associated with an overexpression of cyclin D1 (70 % of cases) [4]. Deregulated expression of cyclin D1 is found in a large variety of malignancies and often associated with tumor progression [5]. Despite activating mutations in β-catenin and overexpression of cyclin D1 SPN are largely benign tumors and the reason for this benign behavior remains elusive. A specific feature correlated with overexpression of cyclin D1 in SPN is the aberrant expression of the transcription factor FLI1 [6]. This transcription factor is well known as one part of the EWS/FLI1 fusion protein, product of the translocation t(11;22)(q24;q12) and defining feature of Ewing sarcomas. A large part of the functional knowledge about FLI1 has been derived from studying this fusion product. The EWS/FLI1 fusion protein largely retains the DNA binding specificity of FLI1 [7, 8] and studying its regulatory function several genes of interest have emerged that may also play a role in SPN.

FLI1 interacts with the Wnt/β-catenin pathway by regulating the expression of the Wnt inhibitor DKK1. In the form of EWS/FLI1 the transcription factor inhibits the expression of DKK1 in Ewing sarcomas [9] but its role in SPN is still unknown. Other members of the Wnt/β-catenin pathway are the BCL9 and BCL9L proteins that may stabilize β-catenin and support its transcription inducing function. The tumor promoting function of both genes has mainly been studied in colon cancer so far [10, 11], but given the known role of the Wnt signaling pathway in pancreatic neoplasias and especially SPN their function in these tumors needs to be investigated. A target gene of FLI1 itself without the fusion partner is INPP5D (SHIP1) [12]. This protein inhibits D-type cyclins, including cyclin D1, in osteoclast precursors in an Akt dependent manner [13]. In concordance with this is an increased expression of p27, also regulated via the Akt pathway. Elevated levels of p27 and p21 have already been demonstrated in SPN [14]. In pancreatic adenocarcinomas the Akt pathway is activated by IGF and the ratio of IGF and its inhibitor IGFBP3, another target of EWS/FLI1 [15], may play a role in the development of pancreatic cancer [16]. Additionally, overexpression of IGFBP3 was found in pancreatic cancer cells [17] and has been shown to promote metastases in pancreatic endocrine neoplasms [18]. The IGF-1 pathway is also critical for the pathogenesis and proliferation of Ewing sarcomas and directly regulated by EWS/FLI1 [19, 20]. In Ewing sarcoma cell lines EWS/FLI1 suppresses expression of IGFBP3 [15], but the transcriptional activity of the wildtype transcription factor FLI1 in SPN may differ. Also involved in the Akt pathway and dependent on IGF signaling is the EWS/FLI1 target PBK (also called TOPK) [21, 22]. PBK phosphorylates and activates Akt and contributes to the degradation of its inhibitor PTEN [23].

The objective of this study was to elucidate the molecular basis for the astonishingly benign behavior of SPN in the face of an activated Wnt pathway and additionally an overexpression of cyclin D1, two factors usually associated with aggressive malignancies. To this purpose the RNA expression of the FLI1 regulated genes DKK1, INPP5D, IGFBP3 and PBK and of FLI1 itself was investigated. Additionally, to further evaluate the Wnt/β-catenin pathway the expression of its members BCL9 and BCL9L was examined. By correlating the RNA expression of the various genes with the expression of FLI1 we further addressed the question of the role of FLI1 overexpression in SPN. Identifying factors that render SPN benign may in reverse shed light on factors underlying the aggressive behavior of PDAC and elucidate avenues to modify that behavior.

## Methods

### Patient samples

Eighteen tumor resection specimens of solid pseudopapillary neoplasms (SPN) of the pancreas were obtained from the consultation files of Prof. Günter Klöppel, former head of the Department of Pathology of the University of Kiel. Additionally 12 tumor resection specimens of pancreatic adenocarcinoma (PDAC) and intraductal papillary mucinous neoplasm (IPMN) each were obtained from the archive of the MVZ Hanse Histologikum in Hamburg. The study was non-interventional and samples investigated in this study were acquired during necessary medical procedures and submitted for clinically indicated diagnostic procedures to this Institute. All samples were anonymized at the start of the study. This country’s (Germany) ethics policies and medical research laws do not require approval by an ethics committee when leftover diagnostic material is used in research in accordance with the Declaration of Helsinki. Written informed consent to use leftover diagnostic material for research purposes was obtained from all patients included in the study that were still alive.

### RNA-Extraction and quantitative real time PCR

Tumor tissue of pancreatic neoplasias was manually microdissected from formalin fixed, paraffin embedded tissue blocks. For IPMN samples, Laser-microdissection was performed because of the rarity of the tumor cells. After microdissection each sample contained at least 75 % of tumor cells. Total RNA was extracted using the RNeasy FFPE kit (Qiagen, Germany). To evaluate the RNA expression of the genes FLI1 (NCBI RefSeq: NM_002017.4), DKK1 (RefSeq: NM_012242.2), INPP5D (SHIP1) (RefSeq: NM_005541.4), PBK (TOPK) (RefSeq: NM_018492.3), IGFBP3 (RefSeq: NM_000598.4), and BCL9 (RefSeq: NM_004326.3) and BCL9L (RefSeq: NM_182557.2) quantitative real time RT-PCR was performed on the StepOne Real Time PCR System (Life Technologies, USA) using the Qiagen OneStep RT-PCR kit (Qiagen, Germany) according to the manufacturer’s instructions. For each PCR 50 cycles were run consisting of 15 sec. at 96 °C and 1 min at 60 °C following an initial reverse transcription step of 30 min at 50 °C and 15 min at 96 °C. TaqMan MGB-probes were used for detection of the PCR product. Primers and probes for each analyzed gene are given in Table 1. Relative expression ratios were calculated according to the formula:

**Table 1:** Primers and probes

Gene	Primers (for/rev)	Probe
GAPDH	TTTGGTATCGTGGAAGGACTC / GAACATCATCCCTGCCTCTAC	CATGCCATCACTGCC
FLI1	CCAGATCCGTATCAGATCCTG / CAACGCCAGCTGTATCACCT	CAGATCCAGCTGTGGCAA
DKK1	GCATGCGTCACGCTATGTGC / TGGTAATGATCATAGCACCTTGG	CTGATCAAAATCATTTCC
INPP5D	ACGGAGCGTGATGAATCCAG / CAGCATCACTGAAATCATCAAC	AAGTCACTAGCAGGGCC
IGFBP3	ACTACGAGTCTCAGAGCACAG / GACACACTGAATCACCTGAAG	ACGGCAGGGACCATA
PBK	TGTTATTACTGACAAGGCAGAC / GATGAAGCATACTATGCAGCG	ATGACTTTATCGATTCCAC
BCL9	CCATGATGCTATCAAGACTGTG / CGAGGATTCTGTGTATTAATGC	CCAGCTCAGATGACGAC
BCL9L	CACAATGCCATCAAGACCATC / AGTTCAGGTGCATCTGGCTG	TCAGACGACGAGCTGC

$$ \mathrm{ratio} = \frac{2^{\varDelta \mathrm{C}\mathrm{P}}{{}_{\mathrm{target}}}^{\left(\mathrm{mean}\ \mathrm{control}\ \hbox{--}\ \mathrm{mean}\ \mathrm{sample}\right)}}{2^{\varDelta \mathrm{C}\mathrm{P}}{{}_{\mathrm{ref}}}^{\left(\mathrm{mean}\ \mathrm{control}\ \hbox{--}\ \mathrm{mean}\ \mathrm{sample}\right)}} $$

using normal pancreatic tissue (NPT) as control tissue and GAPDH as reference gene for normalization. The mean ratio from two independent experiments was used. The NPT control sample was prepared from RNA pooled from nine different patients.

### Statistical analysis

Expression levels of RNA in SPN, PDAC and IPMN were compared to normal pancreatic tissue and between the different tumor entities using Student’s *t*-test. Ratios were normalized and linearized using binary logarithm. Correlations of the expressions of different genes were analyzed using Pearson’s correlation coefficient.

## Results

**Fig. 1:**
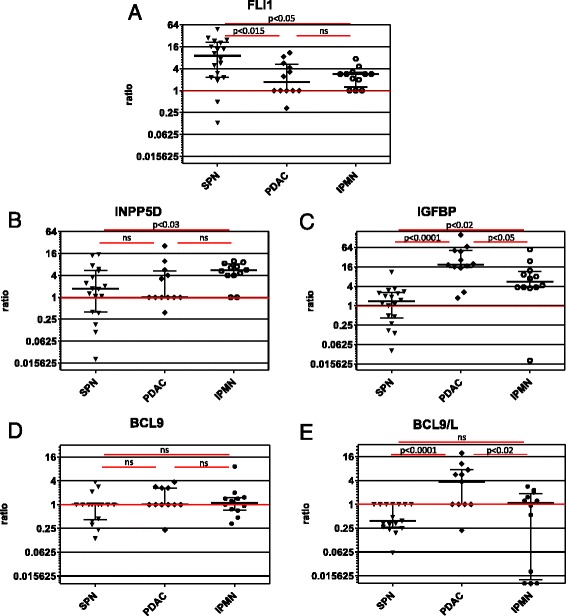
Expression of FLI1, INPP5D, IGFBP, BCL9, and BCL9L in pancreatic tumors. RNA expression of FLI1 (**a**), INPP5D (**b**), IGFBP (**c**), BCL9 (**d**), and BCL9L (**e**) in various pancreatic neoplasias. The expression was normalized against GAPDH and a pool of normal pancreatic tissues (NPT) was used as control sample, rendering the expression in NPT = 1, as demonstrated by the red line. For each entity the median expression is indicated by a black line and error bars indicate the interquartile range. Statistical differences in RNA expression between different pathological entities are shown above the groups and significance levels are indicated as p-values using Student’s *T*-test

Eighteen cases of solid pseudopapillary neoplasms of the pancreas were evaluated for RNA expression levels of FLI1, DKK1, INPP5D (SHIP1), IGFBP3, PBK (TOPK), and BCL9 and BCL9L. Microdissection of the tissue ensured a high proportion of tumor cells (75 % or above) in the investigated sample. The original RNA expression data are provided in Additional file 1. Expression of FLI1 was increased up to 48 fold in all but 3 of the 18 SPN samples (83 %). In 9 samples FLI1 expression was more than 10 fold higher than in normal pancreatic tissue (NPT), making this overall a very clear and highly significant increase (*p* < 0.0001, Fig. 1a). In IPMN FLI1 expression was increased 2–5 fold in 8 out of 12 samples, the increase being significant (*p* < 0.001, Fig. 1a). In PDAC, however, FLI1 expression was only increased in 5 of the 12 samples (42 %). There was no significant difference to FLI1 expression in normal tissue (*p* < 0.08, Fig 1a). FLI1 expression was also significantly higher in SPN than in IPMN or PDAC (*p* < 0.05 and *p* < 0.015 resp.).

No significant difference in the expression levels of INPP5D was found between SPN or PDAC samples and NPT (Fig. 1b). However, a clear and highly significant increase in expression of INPP5D was found in 10 out of 12 samples of IPMN, the increase ranging between 4 and 10 fold (*p* < 0.0001, Fig. 1b). INPP5D expression was also significantly higher in IPMN than in SPN (*p* < 0.03, Fig. 1b), but no significant difference was detected between IPMN and PDAC (*p* < 0.08).

Expression of IGFBP3 was highly increased up to 160 fold in all but one of the PDAC samples, as expected (*p* < 0.00001, Fig. 1c). A similar result was seen for IPMN, where again the expression was increased in all but one case up to 55 fold (*p* < 0.001, Fig. 1c). On the other hand, in SPN no significant increase in the expression of IGFBP3 was found. The differences between the three entities were significant, too, with PDAC showing a higher expression of IGFBP3 than IPMN (*p* < 0.05) and SPN (*p* < 0.0001) and IPMN still demonstrating a higher expression than SPN (*p* < 0.02, Fig. 1c).

BCL9 and BCL9L are very similar genes that are supposed to have a similar function in the Wnt pathway. Nevertheless, they showed differing expression patterns in pancreatic neoplasias. No significantly different expression of BCL9 was found between normal tissue and any of the pancreatic neoplasias investigated (Fig. 1d). BCL9L expression, however, was significantly increased in PDAC (*p* < 0.04, 6 of 11 samples), but significantly decreased in SPN (*p* < 0.001, 10 of 17 samples, Fig. 1e). In IPMN the expression of BCL9L overall showed no significant difference to normal tissue, although some cases demonstrated a strongly reduced expression (Fig. 1e). No correlation was found between the BCL9L ratio and the grade of dysplasia or the histological type of the IPMN (not shown).

Expression levels of PBK were very low in all pancreas tissues (normal and neoplastic), making a meaningful analysis of changes in RNA expression not feasible. Furthermore, DKK1 expression was extremely low in normal pancreatic tissue, but somewhat higher in 11 of 18 (61 %) samples of SPN, 6 of 12 (50 %) samples of IPMN and 10 of 12 (83 %) samples of PDAC. However, the overall very low expression of the DKK1-RNA made a statistical analysis of the ratios unreliable. Nevertheless, the DKK1 expression in any pancreatic neoplasia seems to be higher than in normal tissue.

**Fig. 2:**
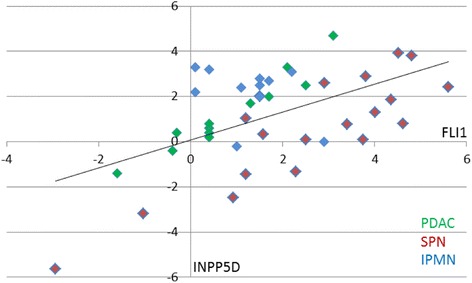
Correlation of the expressions of FLI1 and INPP5D. The RNA expressions of FLI1 and INPP5D correlate well in PDAC (green), and to a lesser extent in SPN (red). In IPMN (blue) a correlation between the two RNA expressions is not detectable. The line indicates the overall correlation

Expression levels of the transcription factor FLI1 in SPN demonstrated a significant positive correlation of a linear type to the expression of INPP5D (*r* = 0.88; *p* < 0.00001, Fig. 2) and IGFBP3 (*r* = 0.84; *p* < 0.00001, not shown), although both these genes did not show significantly increased RNA expression in SPN. In PDAC, however, only FLI1 and INPP5D showed a strong positive correlation (*r* = 0.97; *p* < 0.00001, Fig. 2), although neither gene was highly expressed. No correlation, however, was found between FLI1 and IGFBP3. No correlation between FLI1 and these two genes was detected in IPMN (Fig. 2).

## Discussion

SPN are rare tumors of the pancreas with few cases showing metastatic disease. Over 90 % of cases carry a mutation in CTNNB1, the β-catenin gene, leading to activation of the Wnt signaling pathway. Aberrant protein expression and increased activity of the Wnt pathway has been implicated in the pathogenesis of various neoplasias, including pancreatic adenocarcinoma [2, 3]. In SPN, on the other hand, the β-catenin mutation does not lead to an increased proliferation rate and malignant behavior. The reason still remains to be elucidated. Therefore, in this study, SPN were compared to the aggressively behaving PDAC and furthermore to IPMN. The latter entity behaves less aggressively than PDAC but is more aggressive than SPN depending on the grade of dysplasia and accompanying invasive adenocarcinoma.

A further factor associated with SPN is the transcription factor FLI1. Aberrant protein expression has been demonstrated in 63 % of cases [6]. The overexpression of FLI1 in SPN has been confirmed in this study showing a major increase in FLI1 RNA level in 83 % of cases of SPN compared to only 50 % of PDAC and a much less prominent increase in FLI1 expression levels in IPMN. Functionally, FLI1 is linked to the Wnt pathway by regulating the expression of the Wnt inhibitor DKK1 [9]. In Ewing’s sarcoma the fusion protein EWS/FLI1 inhibits basal and β-catenin induced transactivation of the DKK1 promoter [9]. It has been hypothesized that this decrease in DKK1 expression may contribute to the aggressive and highly malignant behavior of Ewing’s sarcomas. There are also hints of an overexpression of DKK1 in especially aggressive pancreatic adenocarcinomas [24]. The present study, however, did not confirm a high expression of DKK1 in pancreatic tumors. The expression was generally very low in all tumor entities and SPN, too, are no exception. Therefore an inhibition of DKK1 is not verifiable here and most probably does not play a part in the low malignancy of SPN.

The IGF-1 pathway is functionally important for the pathogenesis and progression of Ewing Sarcoma [19, 21] and the fusion protein EWS/FLI1 has been shown to downregulate the expression of IGFBP3 in Ewing sarcoma cell lines [15]. However, an effect of the transcription factor FLI1 on IGFBP expression levels was not confirmed in most pancreatic tissues. In SPN even a highly significant overexpression of FLI1 is not enough to change IGFBP3 levels from those in normal pancreatic tissue. In contrast, PDAC show a very high overexpression of IGFBP3, confirming the results of studies on pancreatic cancer cell lines [17]. This is rather surprising, since high serum levels of IGFBP3 are supposed to inhibit IGF1, thereby reducing the availability of a relevant growth factor for PDAC and the relative levels of IGFBP3 and IGF in serum have been associated with risk of pancreatic cancer, at least in some studies [16, 25]. A possible explanation may be provided by the fact that IGFPB3 seems to be upregulated in pancreatic cancer cells under hypoxic stress [26] and pancreatic xenograft tumors under neoadjuvant therapy [27]. IGFBP3 may be overexpressed due to stressful conditions to downregulate growth and allow the pancreatic cancer cell to survive in an adverse environment with limited resources. In this theory, lack of overexpression of IGFBP3 in SPN and, to a lesser extent, in IPMN may actually be a result and not a cause for the benign behavior of SPN and the less aggressive behavior of IPMN, since the generally slower growth of these tumors can be sustained more easily when resources become limited.

INPP5D is a cyclin D1 inhibitor in osteoclast precursors and also regulated by FLI1 [12]. Cyclin D1 is overexpressed in SPN but obviously functionally at least in part inactive since the Rb protein is not phosphorylated in SPN [14]. Overexpression of a cyclin D1 inhibitor would account for this lack of downstream activity, but INPP5D does not show a significant rise in RNA-expression levels in SPN compared to normal pancreas tissue or PDAC, although its expression seems to be closely correlated with FLI1 expression in both SPN and PDAC. On the other hand, the more common cyclin D1 inhibitors p21 and p27 have already been shown to be expressed in practically all SPN [14]. Therefore, this functional niche may already be occupied in this tumor entity. PDAC, on the other hand, show aberrant and increased expression of cyclin D1 in 70-80 % of cases (reviewed in [28]) and cyclin D1 is functionally active and relevant for cancer growth and proliferation [29, 30]. Accordingly, the cyclin D1 inhibitor INPP5D shows no increased expression in this entity. However, INPP5D is overexpressed in IPMN, a less aggressive form of pancreatic neoplasia. Data on cyclin D1 expression in IPMN are sparse, but its expression seems to be higher in PDAC than in IPMN [31]. Overexpression of the inhibitor INPP5D may factor in generating this difference and possibly even play a role for the less aggressive growth of IPMN. SPN, on the other hand, seem to follow a different route in pathogenesis where INPP5D is not involved. This again demonstrates the general divergence of SPN from ductal derived pancreatic neoplasias. IPMNs are, however, also the only one of the three pancreatic tumor entity investigated where no correlation was found between FLI1 and INPP5D expression. In SPN and PDAC, on the other hand the expression of FLI1 showed a positive correlation to the expression of INPP5D. This is an indication that FLI1 may indeed play a role in the transcription of INPP5D in pancreatic tissues. Nevertheless, in leukemogenesis a negative regulation of INPP5D by FLI1 is described whereas in pancreatic tissue the correlation was positive. Tissue specific modifications in the function of the transcription factor may explain this difference.

BCL9 and BCL9L both are part of the Wnt/β-catenin signaling pathway. Although the evidence for their exact roles is somewhat sketchy and inconsistent, there seems to be a consensus that both genes enhance Wnt-signaling by binding to and increasing β-catenin transcriptional activity. This further leads to increased oncogenic signaling [10, 11, 32]. Nevertheless, most investigations were done on colon cancer or leukemia/lymphoma cell lines and so far evaluations of pancreatic cells concerning these two genes are not known. This study demonstrates no difference in the expression of BCL9 in the various types of pancreatic neoplasias we evaluated compared to normal tissue. BCL9L, however, showed a differential expression in SPN, IPMN, and PDAC. Whereas the gene was clearly overexpressed in the highly aggressive PDAC it also showed a decreased expression in nearly 60 % of the mostly benign SPN. IPMN, of intermediate malignant behavior, demonstrated a high variance in the expression of BCL9L with most cases showing an expression at the level of normal pancreatic tissue. However, a few IPMN cases nearly completely lacked expression of BCL9L. Overall the expression of BCL9L nevertheless correlated with the aggressiveness of the tumor. This is a strong hint that BCL9L may contribute to overactivation of β-catenin in PDAC [33, 34], especially since the overexpression of the Wnt/β-catenin pathway seems to be correlated with increased aggressiveness and exceptionally poor prognosis [24, 35] via stabilization and activation of β-catenin BCL9L may also promote the increased expression of the β-catenin target cyclin D1. On the other hand, a lack of BCL9L expression in SPN may lead to a faster degradation of β-catenin and reduced function in the nucleus [10, 32], thereby preventing the protein from fulfilling its transcriptional, and in this case oncogenic, role.

## Conclusions

This study provides a first clue to the possible reasons for the astonishingly benign behavior of SPN by demonstrating a significantly decreased expression of the β-catenin stabilizing gene BCL9L in this entity. The involvement of the β-catenin gene in the pathogenesis of SPN is already known, because of the high mutation frequency of the gene (over 90 %) [1]. Therefore, an attenuation of β-catenin function is needed to decrease the oncogenic potential of the gene and account for the favorable prognosis of SPN. Moreover, the expression of BCL9L was significantly increased in the aggressively malignant PDAC, making a connection to the lack of malignancy in SPN even more likely. Reasoning from the point of view of PDAC, the high BCL9L expression may in part contribute to its aggressive course. This argument is further strengthened by the generally intermediate position of the IPMN: intermediate in malignant behavior and intermediate in BCL9L expression. On the other hand, IPMN may simply follow a functionally different pathogenetic path. Overexpression of the cyclin D1 inhibitor INPP5D may be involved in the less aggressive growth pattern of IPMN, but this mechanism does not seem to play any role in the benign behavior of SPN. When seen in context with other studies, the high overexpression of IGFBP in PDAC and, to a lesser extent also in IPMN may rather be a secondary event and not contribute directly to the initiation of aggressive malignancy.

## Additional file

Additional file 1: Mean RNA expression Ratio derived from two independent experiments, normalized against GAPDH. (XLSX 12 kb)
